# Unplanned placement changes in children’s homes: an observational study of national administrative data on children in care and providers in England

**DOI:** 10.1136/bmjph-2025-004219

**Published:** 2026-07-01

**Authors:** Rick Hood, Allie Goldacre, Emma Martin, Hannah Tempest, Caroline Coady, Keith Clements, Edward Jones, Rebecca Vincent, Chao Wang

**Affiliations:** 1 Kingston University, Kingston upon Thames, UK; 2 Kingston University Faculty of Health Science Social Care and Education, London, England, UK; 3 OFSTED, London, England, UK; 4 National Children’s Bureau, London, England, UK

**Keywords:** Public Health, Community Health, statistics and numerical data

## Abstract

**Introduction:**

Unplanned placement changes often indicate disruption and breakdown in children’s care arrangements while they are looked after by the state. Children in children’s homes in England are known to have high rates of placement instability, but little is known about unplanned moves and the role that provider characteristics play. This study aimed to identify the factors associated with higher and lower rates of unplanned placement change for children placed in a children’s home in England.

**Methods:**

Secondary analysis was undertaken of national administrative data of all children placed in a children’s home in England between 2019 and 2023, who were continuously in care for at least 1 year during that period. The outcome was an unplanned placement change within the first 12 months. Regression analysis was used to calculate the effects of child-level and provider-level variables.

**Results:**

11 730 children had a total of 16 520 placements during the observation period. An association with higher rates of unplanned placement change was observed for children aged 15–16, children from mixed heritage backgrounds, children assessed in relation to risks outside the home, and children who had already experienced multiple moves. Placements in local authority owned ‘in-house’ children’s homes had lower rates of unplanned change than those in outsourced provision. Higher levels of staff turnover and use of agency staff, lack of a registered manager and a shorter period of operation were all associated with higher rates of unplanned placement change.

**Conclusion:**

Our results shed light on the relevance of provider factors when it comes to placement breakdowns and raise concerns that some of the most vulnerable children in care are being placed in unsuitable forms of provision. However, these findings should be considered in the context of wider systemic problems with sufficiency in the English care system.

**WHAT IS ALREADY KNOWN ON THIS TOPIC**

- Multiple changes of caregiver are known to have an adverse effect on children’s development, and children in children’s homes are more likely to experience instability compared with children in foster care and kinship care. However, there is limited evidence on the dynamic factors affecting the likelihood of a placement breakdown in residential care settings.

**WHAT THIS STUDY ADDS**

- This is the first study focusing on unplanned placement changes for children in children’s homes in England. It advances our knowledge about how characteristics of providers and placements are linked to rates of unplanned moves and contributes to emerging evidence on sufficiency in the care system.

**HOW THIS STUDY MIGHT AFFECT RESEARCH, PRACTICE OR POLICY**

- Reducing the currently high levels of placement breakdown in children’s homes will require better matching and support for children with complex needs, greater attention to workforce stability and reshaping the market for residential care to promote stable, high-quality local provision.

## Introduction

On 31 March 2024, there were 83 630 children ‘looked after’ (CLA) in England, mostly living in out-of-home care.^1^ Local authorities (LAs) hold statutory responsibility for these children, but placements are delivered by a mix of local authority, private and voluntary-sector providers. In 2023–24, two-thirds (67%) of CLA were placed in foster care, 10% in a children’s or secure home and 7% in ‘supported accommodation’ designed for semi-independent or independent living.^1^ The remainder were placed for adoption (2%), with parents (7%) or in ‘other’ settings (5%), some of which remain unregistered or regulated by other bodies.^1^ This study focuses on 11 730 children who were continuously looked after for at least 1 year between 2019 and 2023 and who experienced at least one placement in a children’s home. Since children’s homes constitute a distinct care context characterised by higher levels of complexity and instability, understanding drivers of unplanned moves in this setting has specific policy relevance.

Children’s homes are defined in the *Care Standards Act 2000* and must be registered with Ofsted, which inspects them annually. Compared with the wider care population, children in these homes are typically older, more likely to have experienced multiple placements and more likely to have complex needs.^2^ About 80% have identified special educational needs.^3^ Unlike children in foster care, most remain in care until age 18. The demand for residential placements has grown in recent years as children entering care are, on average, older and have greater and more complex needs.^4^ Many experience poor mental health and limited access to evidence-based treatment.^5^ Older adolescents are particularly vulnerable to child criminal and sexual exploitation. Over the life course, care-experienced people face marked health and social inequalities, with the poorest outcomes among those who spent time in residential care.^6^

Concerns about the sufficiency and quality of care provision in England have been longstanding.^7^,^9^ The number of traditional foster carers has fallen, only partly offset by an increase in kinship carers. The residential care sector faces particular challenges, including high staff turnover, shortages of qualified workers and persistent negative perceptions of residential care as a ‘last resort’ when family placements fail.^10 11^ Low morale and limited professional recognition have further undermined stability and quality. At the same time, increasing marketisation has prompted concerns about profiteering and poor value for money as well as the risk of unsuitable or unsafe placements.^12 13^ Between 2019–20 and 2023–24, local authority spending on residential care almost doubled, rising from £1.6 billion to £3.1 billion.^9^ Yet provision remains unevenly distributed with homes disproportionately located in low-cost housing areas, often far from children’s home communities.^14^ Many children are consequently placed at long distances from family, friends and local services, which can negatively affect their well-being and stability.^9 14^

### Placement stability and breakdown

Placement stability is a key indicator of quality and continuity of care in residential settings.^15 16^ It is typically measured through the number of placement or caregiver changes experienced over time. Frequent changes are associated with poorer emotional and social development. National statistics classify children with three or more placements in a year as experiencing ‘high instability’.^1^ By this measure, around 10% of all CLA and 14% of children in children’s homes experience high instability. However, this metric captures only changes in placement type and does not reflect the turnover of staff or peers within a home, factors that may also undermine a sense of stability and belonging. The international literature, reviewed by Riemersma *et al*,^16^ has tended to focus on children’s demographic characteristics and assessed risks rather than on features of the care environment. Provider factors such as staffing levels, workforce turnover or distance from home are less frequently examined, often due to data limitations. When children experience multiple placements, it becomes difficult to attribute instability to the quality of a specific setting. Nonetheless, these provider-level factors are likely to be crucial in shaping children’s experiences and outcomes.

It is also important to distinguish between *planned* and *unplanned* placement moves. While planned changes may form part of a child’s care pathway, unplanned moves typically signal breakdowns in care arrangements or mismatches between need and provision. Most existing studies focus on these unplanned moves, viewing them as indicators of instability or system failure.^16^ In England, however, national data often aggregate all placement moves, making it difficult to isolate unplanned changes or to examine how provider characteristics influence them. Shortages of appropriate placements, emergency admissions, breakdowns and out-of-area placements reflect both inadequate local capacity and systemic pressures.^17^ These dynamics are shaped by wider factors, including workforce shortages, market concentration and regional inequalities in provision, all of which limit children’s choice and disrupt continuity of care.^14^

Despite policy attention to placement stability, evidence on the drivers of unplanned moves in children’s homes remains limited. National administrative datasets provide an opportunity to examine these dynamics systematically, linking information about children, their care histories and provider characteristics. This study used 5 years of national data to examine which child, care history and provider characteristics were associated with unplanned placement changes within 12 months of entering a children’s home.

## Materials and methods

### Design and setting

This study used a retrospective cohort design based on national administrative data for children placed in a children’s home in England between 2019 and 2023.

### Participants and data sources

The core dataset was the Children Looked After (CLA) return, which records every care episode for children looked after by local authorities (LAs) in England.^18^ This anonymised dataset includes information on each placement’s start and end dates, type and provider, legal status, distance from home, reason for change and child demographics. Provider-level information was appended from Ofsted’s Annex A dataset,^19^ compiled annually during inspections under the Social Care Common Inspection Framework. Annex A contains data on workforce characteristics, including staff turnover, agency worker use, tenure and capacity, covering all registered children’s homes in England. These data were linked to the CLA using Ofsted’s Unique Reference Number (URN), matching the placement start date to the closest inspection date.

We also appended a bespoke dataset on registered manager status, indicating whether each home had a registered manager in post and the duration of tenure, linked via the Ofsted URN. Information on children’s previous social care involvement was drawn from the Children in Need (CIN) census using a concatenated LA–child identifier common to both datasets. The CIN dataset includes prior assessments and child protection plan status. A full variable list and data summary are provided in the supplementary table.

### Study cohort

The study population comprised all children’s home placements beginning between 1 April 2019 and 31 March 2023, where the observation period for placement change was at least 1 year (n=16 360), that is, the ‘exposure’ or ‘risk’ period was 12 months minimum. Placements for young people aged 17 who exited care within a year (because of ageing out or early exit) were excluded, as were respite placements, which are planned and cyclical by design. The follow-up period extended to 31 March 2024.

Placements that could not be matched to the Annex A dataset or lacked workforce data were analysed separately, given their potential for bias. A small number of records with missing data on key model variables were excluded. Details of inclusion and exclusion criteria, and a flow diagram of the cohort selection process, are provided in online supplemental table 1.

### Definition of placement change

A placement change was defined as a change in placement and carers, or a simultaneous change in legal status and carers, identified through the CLA field reason for new episode (codes P and B). Each placement change was categorised as planned, unplanned or unknown according to the variable reason for placement change. Twelve distinct reasons were recorded in the CLA; these were recoded into three groups:

- Planned – change forming part of the child’s care plan
- Unplanned – change not part of the care plan, typically indicating placement breakdown
- Unknown – recorded as ‘other’ or unclassified

For descriptive analysis, subcategories of unplanned changes are shown separately in online supplemental table 3. Due to small cell counts, unplanned moves were analysed as a single group in regression models. Coding for placement changes is listed in online supplemental table 6.

### Statistical analysis

The primary outcome was the occurrence of an unplanned placement change within 12 months of the placement start date. We first calculated descriptive statistics showing the proportion of placements ending in change overall and by category (planned, unplanned or unknown) for each child and care characteristic. In total, 50% of placements ended in change within 12 months: 18% were planned, 19% unplanned and 13% unknown.

Variables were conceptualised across three domains:

- Child characteristics (eg, age, gender, ethnicity and disability)
- Care and provision prior to placement (eg, number of prior moves, legal status and prior protection plan)
- Care and provision during placement (eg, provider type, distance from home and workforce indicators)

**Figure 1:**
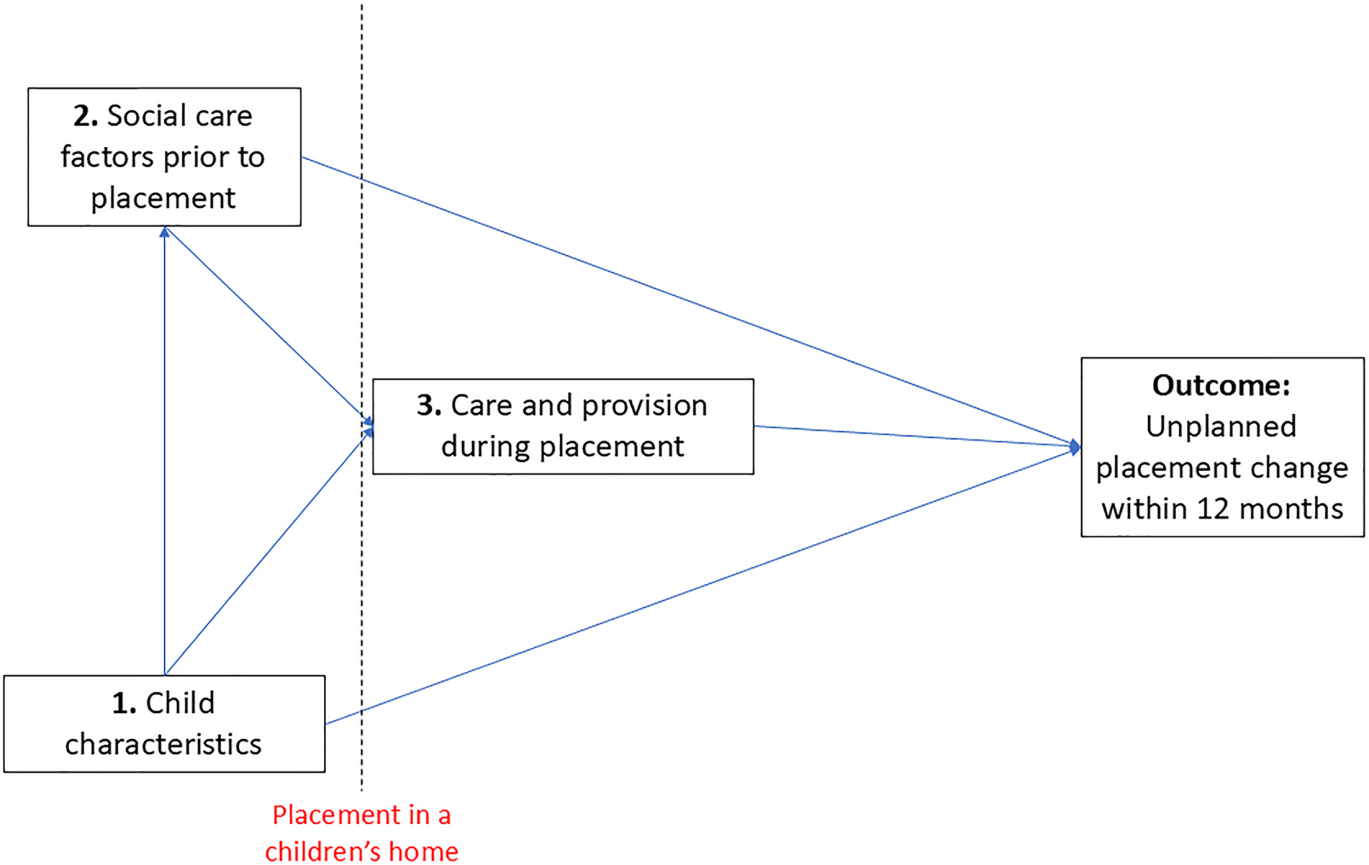
Theoretical pathway linking child and provider factors to unplanned placement change.

A simplified logic model (figure 1) guided the analysis. Child characteristics could influence stability directly or indirectly through care history. Provision prior to placement might also affect provision following placement, which in turn influences the outcome. Statistically, the relationship between post-placement provision and stability may therefore be confounded by earlier variables.

Stable placements (ie, placements with no recorded change within 12 months of the start date) were used as the reference category, allowing estimation of the relative probability of planned, unplanned or unknown placement changes within the follow-up period. Placement changes occurring after 12 months were treated as ‘no change within follow-up’ for the primary outcome. We used multinomial logistic regression to model the probability of each outcome (planned, unplanned, unknown) occurring within 12 months of the placement starting. The models were progressively adjusted for potential confounders based on the theoretical framework. As some children contributed more than one placement during the observation period, cluster robust standard errors at the child level were used to account for within-child correlation between observations. Predictive margins derived from model coefficients were used to estimate the adjusted probability of an unplanned change within 12 months, presented as percentage point differences with 95% CIs in forest plots. Metrics for model performance, including pseudo (McFadden’s) R-squared, the Akaike Information Criterion and Bayesian Information Criterion, as well as the overall likelihood-ratio test, are presented in online supplemental table 5.

Interaction terms were specified a priori based on the study’s conceptual framework and policy relevance to explore whether associations varied across strata of key indicators. For instance, placement ownership (in-house vs outsourced) was combined with placement location (within or outside the placing LA) and distance from home, enabling assessment of heterogeneity across all three variables. Workforce variables, that is, staff turnover and agency worker usage, were also combined to capture compounding effects of instability in staffing.

Analyses were conducted using Stata 18. The primary focus was on unplanned placement changes; results for planned and unknown changes are reported in online supplemental table 3 for completeness.

The proportion of unmatched or missing records in linked datasets was relatively small; therefore a complete-case analysis was used. In large datasets, this approach provides unbiased estimates when missingness depends on observed covariates rather than the outcome, with minimal loss of efficiency (Carpenter & Kenward, 2013).

### Data quality and linkage

The linkage between the CLA and Annex A datasets achieved a 90% match rate. Unmatched placements were slightly more likely to end unplanned (22.2% vs 19.5%), which suggests potential but limited selection bias. Prior care history linkage via the CIN census was limited to placements where children had remained within the same LA, since identifiers are not transferable across authorities. These constraints are discussed further in the limitations section.

### Patient and public involvement

The study benefited from two experts by experience advisory groups: one group of young adult care leavers and one group of parents with experience of CSC services. Consultation and engagement sessions with these groups have informed our topic of research, priority areas for analysis and interpretation of findings. The results from this work will be used to feed into our ongoing engagement work with policymakers, practitioners, families, young people and the wider community of interest.

### Ethics and data protection

The study received ethical approval from Kingston University Research Ethics Committee (Ref 3469) and research governance authorisation from Ofsted and the Department for Education. Data were accessed and analysed within a secure restricted environment on Ofsted servers. All outputs were subject to statistical disclosure control, including rounding and suppression of small cell counts (<10).

## Findings

### Cohort description

The cohort consisted of 11 730 children, who during the study’s observation window had a total of 11 750 periods of care comprising 16 520 placements. All these children were continuously in care for at least 1 year between 2019 and 2023, with at least one placement in a children’s home starting before the age of 17. The unit of analysis was placement in a children’s home and so descriptive statistics are reported at the placement level, that is, children who had more than one children’s home placement were captured at different time points. 27.1% of children’s home placements started when children were aged 12 years or younger and 37.5% of placements started when children were 15 or 16 years old. Gender was only available as a binary male/female category, with a higher proportion of placements for male children (58.7%). Only broad classifications were recorded for ethnicity, with four-fifths of children’s home placements made for children from a White background (79.4%), compared with 6.9% for Black children and 10.5% for children of mixed heritage.

Children’s placement history varied, with only 17.7% of placements in a children’s home recorded as the child’s first. 31.7% of placements were for children who had previously been in a children’s home, whereas 37.4% had previously been in a foster home. About a third (32.8%) of children’s home placements were made for children whose prior placement was recorded as ‘unplanned’, and over two-fifths (43.4%) were for children who had already had four or more prior placements. The majority of children (62.5%) had received a social care assessment within 2 years of their placement starting. For children in these placements, the most common categories of need were risks in and outside the home (12.2%), child’s mental health (10.5%) and risks outside the home (7.9%). About a fifth of children (20.5%) had been on a child protection plan within 2 years of starting their placement. Over half (52.7%) were placed under a full care order at the point their placement started, with 30.3% placed under a voluntary ‘Section 20’ arrangement and the remainder mainly subject to ‘interim’ care orders (which can be made while court proceedings are still ongoing). A full breakdown of cohort characteristics is provided in online supplemental table 2.

### Provider characteristics

Provider characteristics are also described at the placement level, that is, children’s homes that provided more than one placement during the observation period were captured at different time points. The most common type of placement was a private or voluntary sector provider, outside the LA boundary, more than 50 miles from the child’s original home – this was the experience of a quarter of children placed in a children’s home (24.9%). Only 18.3% of placements were for ‘in-house’ children’s homes (owned and run by the local authority) within the LA boundary. In terms of capacity and occupancy, over half of all placements (55.3%) were in four-bed to six-bed children’s homes, with 31.2% in smaller (1–3 bed) homes and 14.6% in homes with more than seven beds. In 15.2% of placements, the child was the sole occupant of the home during their placement. About three out of every ten placements (29.6%) were in a children’s home with no registered manager in place at the start of the placement. Workforce data was only available for placements after 2019; about half of these placements (49.7%) were in homes that used agency staff and 28.2% were in homes that recorded over 50% staff turnover. One third of placements (33.3%) were in children’s homes that had been operating for 10 or more years, with 18.6% of placements in homes that had opened during the previous year.

### Rates of placement change

**Figure 2:**
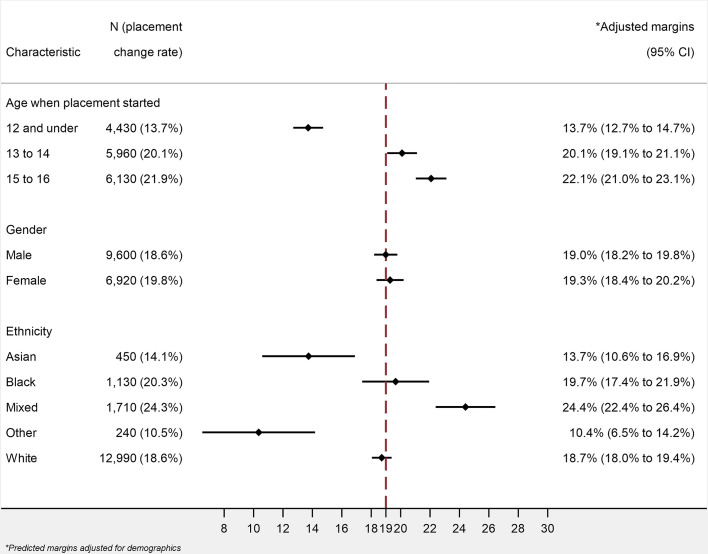
Forest plot showing associations between child characteristics and rates of unplanned placement changes within 12 months of entering a children’s home.

Half of all children’s home placements (49.7%) ended with a change of placement within 12 months of the start date. Just under a fifth (19.1%) ended with an unplanned change, a slightly higher rate than a planned change (18.2%). Unplanned changes were mainly the result of the provider requesting an end due to the child’s behaviour (12.2%). The highest rates of unplanned placement changes were reported for children already experiencing high levels of instability, that is, children who already had four or more placements *and* whose previous placement had ended in an unplanned change; placements for children with this profile saw a higher rate of unplanned change if the prior placement was in a children’s home (36.6%) or another residential setting (38.9%) rather than a foster home (20.2%). The lowest rates of unplanned placement changes were for children accommodated in a larger children’s home with six or more occupants during the placement (11.3%). Variation in planned placement changes differed in some respects from unplanned changes. For example, placements in an in-house children’s home within the LA boundary saw relatively high rates of *planned* changes (30.4%) but relatively low rates of unplanned changes (11.6%). Unplanned placement change rates for all cohort and provider characteristics are shown in figures2^4^ below. Rates for planned changes are reported in online supplemental table 3) along with the breakdown in reasons for unplanned moves.

**Figure 3:**
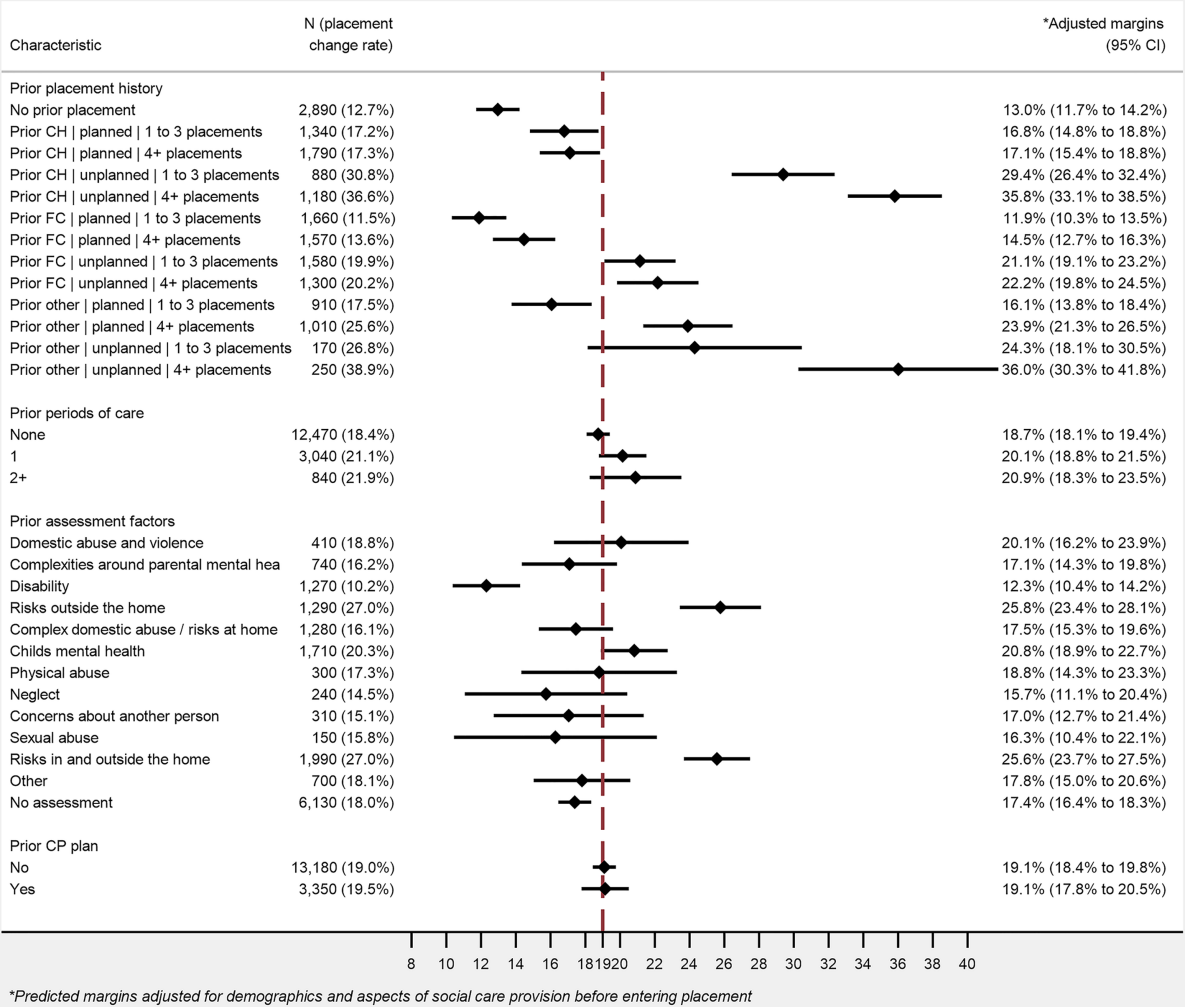
Forest plot showing associations between aspects of prior care and provision and rates of unplanned placement changes within 12 months of entering a children’s home. CH, children's home; CP, child protection; FC, foster care.

**Figure 4:**
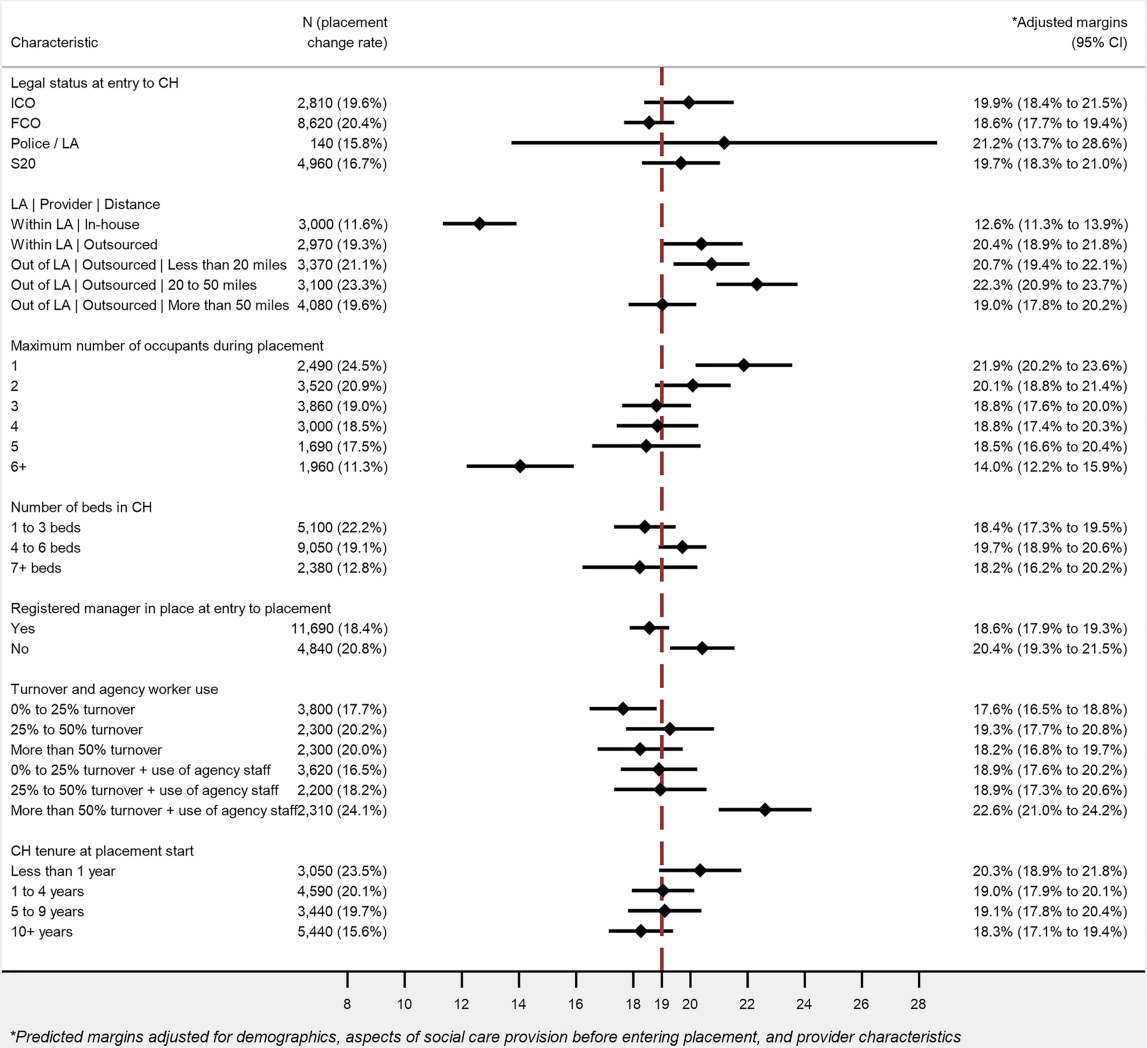
Forest plot showing associations between provider characteristics and rates of unplanned placement changes within 12 months of entering a children’s home. FCO, full care order; ICO, interim care order.

### Model summary and interpretation

Multinomial logistic regression models were used to examine associations between child, care history and provider characteristics and placement outcomes using stable placements (no change within 12 months) as the reference category. Results are presented as predictive margins showing adjusted probabilities of an unplanned placement change within 12 months of a children’s home placement starting. Full model estimates are provided in online supplemental table 4.

### Child characteristics

Figure 2 shows adjusted probabilities of an unplanned placement change within 12 months, derived from the multinomial logistic regression model. The forest plot presents predictive margins for each characteristic, with points indicating the estimated probability and horizontal lines showing 95% CIs relative to the overall predicted rate. The vertical dashed line in the centre indicates the overall predicted rate, which was 19% of all placements. Points to the left of the line indicate a lower than average rate of placement change and points to the right of the line indicate a higher than average rate of placement change. For each characteristic, descriptive statistics on the number and average rates of unplanned change are shown on the left, and adjusted marginal effects are shown on the right with CIs in brackets.

The results show that after placement in a children’s home, the rate of unplanned placement change tended to be higher for 15–16 year olds and children of Mixed ethnicity. In contrast, under-12 year olds and children from Asian or Other ethnic backgrounds tended to have lower rates of unplanned changes. Little difference was observed in the margins between male and female children. The rates of unplanned placement changes among males and females were broadly similar.

### Care and provision prior to placement

Figure 3 shows adjusted probabilities of an unplanned placement change associated with aspects of care history prior to entering a children’s home. Estimates represent predictive margins from the regression model controlling for child characteristics, with 95% CIs shown for each category.

Children with no prior placements or who had moved as a planned change from foster care had much lower rates of unplanned placement change than children who had previously experienced multiple placement moves and/or whose placement began as a result of an unplanned move from another residential setting. Higher rates of unplanned placement change were also seen for children who had multiple periods of care, but this tended to make less of a difference than placement moves experienced within a period of care. Little difference was found in rates of unplanned placement change between children who had been on a CP plan within 2 years of starting a children’s home placement and those who had not. A child’s assessed needs did make a difference, however, based on the categories developed by Hood *et al*.^20^ The highest rates of unplanned placement change were observed for children whose assessments identified concerns about ‘risks outside the home’ and ‘risks in and outside the home’. The lowest rates of unplanned placement change were observed for children whose assessments identified concerns about disability.

### Care and provision following placement

Figure 4 shows adjusted probabilities of an unplanned placement change associated with provider and placement characteristics. Results represent predictive margins from the fully adjusted regression model.

Legal status at entry to a children’s home made a difference, with placements for children on a full care order (FCO) showing lower rates of unplanned moves than children on interim care orders (ICO) or those accommodated under a voluntary ‘Section 20’ arrangement with parents. The highest rates of unplanned changes were for children placed in a children’s home under police protection, which is usually only done in an emergency.

Some provider characteristics were associated with substantial differences in the rate of unplanned placement change. Placements in LA in-house children’s homes were associated with a much lower rate of unplanned moves than placements in outsourced children’s homes. Greater distance from the child’s home before entering care was associated with higher rates of unplanned changes, although outsourced placements more than 50 miles away had lower rates of unplanned change than other outsourced settings. A higher number of occupants during placement was progressively associated with lower rates of unplanned moves. The pattern was not quite as clear for the number of beds a children’s home was registered for, while the lowest rates were associated with the largest homes (7+beds), the highest rates were for four-bed to six-bed homes. The extent of staff turnover and agency workers in children’s homes was associated with variation in unplanned placement changes, with the lowest rates in homes with low turnover and no agency staff and the highest rates in homes with high turnover and use of agency staff. The presence of registered manager also made a difference, with higher rates of unplanned placement changes associated with homes where there was no manager in place at the start of the placement. Finally, there was a tendency for homes that had been in operation for a longer period of time to have lower rates of unplanned placement changes.

It is worth noting that associations for planned placement moves (and those categorised as ‘unknown’) differed in some respects from unplanned moves. For example, in-house children’s homes showed higher rates of planned but lower rates of unplanned moves (compared with outsourced providers). Information on planned and unknown placement moves is included in online supplemental table 3.

## Discussion

### Strengths and limitations

Several limitations should be considered when interpreting these findings. First, the study uses observational administrative data and therefore identifies associations rather than causal relationships. Although the regression models adjusted for a wide range of child, placement history and provider characteristics, residual confounding remains possible because some relevant factors are not captured in national datasets, for example, the severity of behavioural difficulties, particular therapeutic models used in homes or information on staff training. Second, the analysis was conducted at the level of placements, and because some children experience multiple placements, the dataset includes repeated observations for these children. While this enables examination of placement-level characteristics, it also means observations are not fully independent and may partly reflect the characteristics of children who experience frequent moves. Third, the study relies on administrative records compiled by different LAs that may vary in their recording practices. In particular, coding of the reason for placement change may differ between providers and authorities, creating potential inconsistency in whether moves are categorised as planned, unplanned or unknown. Fourth, linkage between datasets introduces additional constraints. Workforce data from Ofsted’s Annex A dataset could be matched to around 90% of placements, and Children in Need (CIN) data could only be linked where children remained within the same LA. Consequently, some placements and prior care episodes may not be fully captured. Finally, the analysis examined whether an unplanned placement change occurred within 12 months of the start of a placement. This fixed follow-up window does not capture variation in the timing of moves within that period or changes occurring later.

Despite these limitations, the study draws on comprehensive national administrative data covering all children’s home placements in England over a 5-year period, enabling a detailed examination of how child characteristics, care histories and provider factors are associated with placement instability.

Comparison with the international literature shows both parallels and contrasts. As in Riemersma *et al*.’s review,^16^ we found no association between placement instability and the child’s sex or gender. However, while their review reported no differences by ethnicity, our findings identified marked disparities, for example, between children of Mixed and Asian heritage. Consistent with some international evidence,^15 21^ older children were at higher risk of placement breakdown. Our results also showed associations between instability and several ‘static’ factors, such as prior breakdowns, legal status and assessed needs, findings that are not consistently reflected in other studies.^18 19^ In England, older teenagers facing extra-familial risks and those with multiple prior placements were particularly likely to experience unplanned moves.

However, it would be misleading to focus solely on children’s characteristics. Provider factors and the wider market context exerted substantial influence even after adjusting for individual characteristics and aspects of care history. Unplanned moves were least frequent in LA–run (‘in-house’) homes and higher in private and voluntary-sector homes, including those located within the same LA boundary. Greater distance from the parental home was generally associated with instability, which is consistent with research in other jurisdictions.^21 22^ However, the type of ownership appeared more influential than distance from home. This aligns with emerging evidence linking for-profit outsourcing to out-of-area placements, instability^22^ and poorer inspection outcomes.^12 23^ The findings therefore add to concerns about the impact of marketisation on the quality and suitability of residential provision in England.^14^

Workforce characteristics also mattered. Homes lacking a registered manager or experiencing high staff turnover and reliance on agency workers had markedly higher rates of unplanned moves. Previous studies have highlighted the importance of workforce stability for consistent, trusting relationships between staff and children, which is a cornerstone of quality care.^24^,^26^ We are unaware of prior research specifically linking the presence of a registered manager or the length of time a home has been in operation to placement stability. These results are particularly concerning given ongoing recruitment and retention challenges and suggest that rapid expansion of newer providers, particularly in the private sector, may not improve quality in a market already characterised by sufficiency problems, excessive profit-making and regional mismatch between supply and demand.

Compared with the international evidence base, our findings reveal stronger associations between instability and factors, such as ethnicity, assessed needs and prior care history. This suggests that systemic issues in England may amplify existing vulnerabilities. The rise in outsourcing, geographical dispersal of children, managerial weaknesses, workforce churn and the proliferation of new, inexperienced providers all appear to compound instability. These problems should not be interpreted as evidence that children’s homes are inherently unsuitable or only appropriate as a ‘last resort’ for children who cannot be placed with a family. This kind of rhetoric obscures the deeper issue of sufficiency. In a system with too few suitable placements, children with complex needs are more likely to be placed in settings unable to meet those needs, perpetuating a cycle of disruption.

The dynamics of insufficiency are similar to other patterns of ‘inverse care’ in public health, in which access to services varies inversely with need.^27^ In England, such inequalities are evident across multiple public services, from medical care to education and social support.^28^ When it comes to children’s homes, high rates of unplanned placement moves are an indicator of inverse care because they disproportionately affect some of the most vulnerable children in the care system despite the high level of spending on residential care. Children most exposed to extra-familial risks and harms were the most likely to be placed far from home, experience instability and have placements break down. Recent growth in supported accommodation for 16- and 17-year-olds, which lacks the same regulatory protections as children’s homes, may further entrench this pattern if it ends up as a form of default provision for young people who may not be ready for semi-independent living.

Addressing these issues will require coordinated policy action. Planned legislative changes may strengthen Ofsted’s powers to fine unregistered providers and improve transparency of ownership structures. However, stronger measures will be needed to reverse the long-term shift towards marketisation and restore LA capacity for in-house provision. Investment in training, qualifications and evidence-based models of residential care is also crucial. A promising UK example is the *No Wrong Door* model of multi-disciplinary residential hubs, now being implemented by several LAs, which integrates care, education and mental health support within a single team.^29^ Welfare-oriented approaches to extra-familial risks, such as contextual safeguarding, have set out to reform multi-agency systems to prioritise children’s needs while addressing the environments in which harm occurs.^30^

Ultimately, improvements in the quality and stability of residential care cannot be sustained without tackling the underlying drivers of demand. Rising numbers of children entering care reflect wider social and structural inequalities, such as poverty, family stress, inadequate community support and gaps in early help services. Without broader reforms to address these upstream factors, instability will continue to manifest downstream in the care system, with an adverse impact on outcomes for children and young people.

## Conclusion

Unplanned placement changes are a key indicator of disruption in the care system. Our analysis of 16 520 children’s home placements in England (2019–2023) identified multiple sources of variation in unplanned moves, notably ownership type, workforce stability, distance from home and home longevity. These results reinforce the importance of provider-level factors in placement stability and highlight how marketisation, workforce challenges and systemic insufficiency shape children’s experiences of care. Reducing placement instability will require rebuilding local provision, strengthening regulation and workforce capacity and addressing the social conditions that drive children into care.

## Supplementary material

10.1136/bmjph-2025-004219 online supplemental file 1

## Data Availability

No data are available.

## References

[1] Department for Education (2023). Children looked after in england including adoption: 2023 to 2024. https://explore-education-statistics.service.gov.uk/find-statistics/children-looked-after-in-england-including-adoptions/2024.

[2] Schoenwald E, Smyth E, Gwyther J (2022). Understanding Residential Care for Children in Care in England: Analysis of Administrative Data.

[3] Ofsted (2021). The education of children living in children’s homes. https://www.gov.uk/government/publications/the-education-of-children-living-in-childrens-homes/the-education-of-children-living-in-childrens-homes.

[4] Hood R, Goldacre A, Martin E (2025). What factors are associated with placement instability for children in children’s homes in England? A retrospective cohort analysis using national administrative data. The British Journal of Social Work.

[5] Hillier R, Duschinsky R, Fearon P (2025). Increasing Access to Evidence-Informed Mental Health Service Provision for Children in Care in England – National Recommendations for Change.

[6] Sacker A, Murray E, Lacey R (2021). The Lifelong Health and Wellbeing Trajectories of People Who Have Been in Care.

[7] Thomas C (2018). Care crisis review: factors contributing to national increases in numbers of looked after children and applications for care orders. https://www.frg.org.uk/involving-families/reforming-law-and-practice/care-crisis-review.

[8] Suh E, Holmes L (2020). Review of Sufficiency Strategies in London.

[9] National Audit Office (2025). Managing Children’s Residential Care.

[10] Abraham L, Elgie S, Soares V (2022). A qualitative study of the views and experiences of those working in residential children’s homes. Scottish Journal of Residential Child Care.

[11] Holmes L, Connolly C, Mortimer E (2018). Residential Group Care as a Last Resort: Challenging the Rhetoric. *Residential Treatment for Children & Youth*.

[12] Bach-Mortensen AM, Goodair B, Barlow J (2023). For-profit outsourcing and its effects on placement stability and locality for children in care in England, 2011–2022: A longitudinal ecological analysis. *Child Abuse & Neglect*.

[13] Child Safeguarding Practice Review Panel (2022). Safeguarding children with disabilities and complex health needs in residential settings: phase 1 report.

[14] Competition and Markets Authority (CMA) Children’s social care market study: final report 2022. https://www.gov.uk/cma-cases/childrens-social-care-study.

[15] Asif N, Breen C, Wells R (2024). Influence of placement stability on developmental outcomes of children and young people in out-of-home care: Findings from the Pathways of Care Longitudinal Study. *Child Abuse & Neglect*.

[16] Riemersma Y, Harder A, Zijlstra E (2023). Static and dynamic factors underlying placement instability in residential youth care: A scoping review. Child Youth Serv Rev.

[17] Ofsted (2022). How local authorities plan for sufficiency: children in care and care leavers.

[18] Children looked after by local authorities in England (2019). Guide to the SSDA903 collection 1 April 2019 to 31 March 2020 – version 1.3. https://www.gov.uk/government/publications/children-looked-after-return-2024-to-2025-guide.

[19] Ofsted (2017). Children’s homes: inspection forms. https://www.gov.uk/government/publications/childrens-homes-inspection-documents.

[20] Hood R, Goldacre A, Jones E (2023). Categorising Demand for Child Welfare Services Using Latent Class Analysis: A Study of the National Data-sets on Children in Need in England. The British Journal of Social Work.

[21] Cashmore J, Wulczyn F (2024). Pathways of Care: A longitudinal study of children in care in Australia: Introductory article for special issue on Pathways of Care Longitudinal Study. Child Abuse Negl.

[22] Sallnäs M, Vinnerljung B, Kyhle Westermark P (2004). Breakdown of teenage placements in Swedish foster and residential care. *Child & Family Social Work*.

[23] Bach-Mortensen AM, Goodair B, Barlow J (2022). Outsourcing and children’s social care: A longitudinal analysis of inspection outcomes among English children’s homes and local authorities. *Social Science & Medicine*.

[24] Egelund T, Vitus K (2009). Breakdown of care: the case of Danish teenage placements. Int J Soc Welfare.

[25] Bolinger J, Mendes P, Flynn C (2021). Stability in residential care in NSW, Australia: The role of the workforce. Scottish Journal of Residential Child Care.

[26] Ofsted (2024). How local authorities and children’s homes can achieve stability and permanence for children with complex needs. https://www.gov.uk/government/publications/good-decisions-children-with-complex-needs-in-childrens-homes/how-local-authorities-and-childrens-homes-can-achieve-stability-and-permanence-for-children-with-complex-needs.

[27] Tudor Hart J (1971). The inverse care law. The Lancet.

[28] Hood R (2023). Inequality and Social Work.

[29] Collyer H, Hennessey A, Sanders M (2021). Strengthening Families, Protecting Children: No Wrong Door.

[30] Firmin C, Lloyd J (2023). Contextual Safeguarding: The next Chapter.

