# Supplementary material for: Unplanned placement changes in children’s homes: an observational study of national administrative data on children in care and providers in England
Source: BMJ Public Health. 2026 Jul 1;4(3):e004219. doi: 10.1136/bmjph-2025-004219 (PMC13331234; doi:10.1136/bmjph-2025-004219)
Supplement: online supplemental file 1 [file bmjph-4-3-s001.docx]

**Supplementary tables**

**Table 1. Flowchart of cohort selection / inclusion and exclusion criteria**

|  |  |  |  |
| --- | --- | --- | --- |
|  | 903 dataset [March 31st 2024] N = 3,592,390 | \|  \| \| --- \| | Excluding episodes which 1) are not ongoing and do not have a recorded end date; 2) share the same episode LA, child ID, start date, reason for new episode, legal status; 3) part of a period of care that does not have a known start date N [excluded] = 38,750 |
|  | \|  \| \| --- \| |  |  |
|  | Following data cleaning  N = 3,553,640 | \|  \| \| --- \| | Excluding episodes of respite care (reason for new episode codes 'V3' and 'V4') N [excluded] = 410,840 |
|  | \|  \| \| --- \| |  |  |
|  | Following exclusion of respite care  N = 3,142,800 | \|  \| \| --- \| | Aggregating (merging) adjoining episodes that are not changes of placement and carer(s) N [excluded] = 576,110 |
|  |  |  |  |
|  | Following aggregation (merging) of adjoining episodes N = 2,566,690 | \|  \| \| --- \| | Filtering by children's home placements that started for children aged 0 to 16 who were continuously in care for at least one year between 01/04/2019 and 31/03/2023  N [excluded] = 2,550,170 |
|  |  |  |  |
|  | Study population N Children = 11,730 N POC = 11,750 N Placements = 16,520 |  |  |
|  |  |  |  |

**Table 2: Cohort characteristics**

All children's homes placements that started between 01/04/2015 and 31/03/2023 where the risk period of a placement change is at least one year. Placements starting for children aged 17 who exited care within one year (due to aging-out of care) and other early exits from care were excluded. The data is restricted to placements starting before 31/03/2023 but full follow-up (for the outcome of a placement change) is known up until 31/03/2024 across all cases.

| **Characteristic** | **Count** | **Column %** |  |
| --- | --- | --- | --- |
|  |  |  |  |
|  |  |  |  |
| Total | 16,357 | 100.0% |  |
| **Demographics** |  |  |  |
| Age when placement started |  |  |  |
| 12 and under | 4,430 | 27.1% |  |
| 13 to 14 | 5,960 | 36.4% |  |
| 15 to 16 | 6,130 | 37.5% |  |
| Gender |  |  |  |
| Males | 9,600 | 58.7% |  |
| Females | 6,920 | 42.3% |  |
| Ethnicity |  |  |  |
| Asian | 450 | 2.8% |  |
| Black | 1,130 | 6.9% |  |
| Mixed | 1,710 | 10.5% |  |
| Other | 240 | 1.5% |  |
| White | 12,990 | 79.4% |  |
| **Prior care characteristics** |  |  |  |
| Prior placement history |  |  |  |
| No prior placement | 2,890 | 17.7% |  |
| Prior CH \| planned \| 1 to 3 placements | 1,340 | 8.2% |  |
| Prior CH \| planned \| 4+ placements | 1,790 | 10.9% |  |
| Prior CH \| unplanned \| 1 to 3 placements | 880 | 5.4% |  |
| Prior CH \| unplanned \| 4+ placements | 1,180 | 7.2% |  |
| Prior FC \| planned \| 1 to 3 placements | 1,660 | 10.1% |  |
| Prior FC \| planned \| 4+ placements | 1,570 | 9.6% |  |
| Prior FC \| unplanned \| 1 to 3 placements | 1,580 | 9.7% |  |
| Prior FC \| unplanned \| 4+ placements | 1,300 | 7.9% |  |
| Prior other \| planned \| 1 to 3 placements | 910 | 5.6% |  |
| Prior other \| planned \| 4+ placements | 1,010 | 6.2% |  |
| Prior other \| unplanned \| 1 to 3 placements | 170 | 1.0% |  |
| Prior other \| unplanned \| 4+ placements | 250 | 1.5% |  |
| Prior periods of care |  |  |  |
| None | 12,470 | 76.2% |  |
| 1 | 3,040 | 18.6% |  |
| 2+ | 840 | 5.1% |  |
| Assessment factors prior to CH placement |  |  |  |
| Domestic abuse and violence | 410 | 2.5% |  |
| Complexities around parental mental hea | 740 | 4.5% |  |
| Disability | 1,270 | 7.8% |  |
| Risks outside the home | 1,290 | 7.9% |  |
| Complex domestic abuse / risks at home | 1,280 | 7.8% |  |
| Childs mental health | 1,710 | 10.5% |  |
| Physical abuse | 300 | 1.8% |  |
| Neglect | 240 | 1.5% |  |
| Concerns about another person | 310 | 1.9% |  |
| Sexual abuse | 150 | 0.9% |  |
| Risks in and outside the home | 1,990 | 12.2% |  |
| Other | 700 | 4.3% |  |
| No assessment within two years of placement starting | 6,130 | 37.5% |  |
| CP plan within two years of period of care starting |  |  |  |
| No | 13,180 | 80.6% |  |
| Yes | 3,350 | 20.5% |  |
| **Care and provider characteristics at entry to placement** |  |  |  |
| Legal status at entry to CH |  |  |  |
| ICO | 2,810 | 17.2% |  |
| FCO | 8,620 | 52.7% |  |
| Police / LA | 140 | 0.9% |  |
| S20 | 4,960 | 30.3% |  |
| Distance placed and LA |  |  |  |
| Within LA \| In-house | 3,000 | 18.3% |  |
| Within LA \| Outsourced | 2,970 | 18.2% |  |
| Out of LA \| Outsourced \| Less than 20 miles | 3,370 | 20.6% |  |
| Out of LA \| Outsourced \| 20 to 50 miles | 3,100 | 19.0% |  |
| Out of LA \| Outsourced \| More than 50 miles | 4,080 | 24.9% |  |
| Maximum number of occupants during placement |  |  |  |
| 1 | 2,490 | 15.2% |  |
| 2 | 3,520 | 21.5% |  |
| 3 | 3,860 | 23.6% |  |
| 4 | 3,000 | 18.3% |  |
| 5 | 1,690 | 10.3% |  |
| 6+ | 1,960 | 12.0% |  |
| Number of beds (based on closest in-year provider snapshot after child's entry to CH) |  |  |  |
| 1 to 3 beds | 5,100 | 31.2% |  |
| 4 to 6 beds | 9,050 | 55.3% |  |
| 7+ beds | 2,380 | 14.6% |  |
| Registered manager in place at entry to placement (new manager registered within 90 days) |  |  |  |
| Yes | 11,690 | 71.5% |  |
| No | 4,840 | 29.6% |  |
| Turnover and agency worker use (data for post-2019 only) |  |  |  |
| 0% to 25% turnover | 3,800 | 23.2% |  |
| 25% to 50% turnover | 2,300 | 14.1% |  |
| More than 50% turnover | 2,300 | 14.1% |  |
| 0% to 25% turnover + use of agency staff | 3,620 | 22.1% |  |
| 25% to 50% turnover + use of agency staff | 2,200 | 13.4% |  |
| More than 50% turnover + use of agency staff | 2,310 | 14.1% |  |
| CH tenure at placement start |  |  |  |
| Less than 1 year | 3,050 | 18.6% |  |
| 1 to 4 years | 4,590 | 28.1% |  |
| 5 to 9 years | 3,440 | 21.0% |  |
| 10+ years | 5,440 | 33.3% |  |

**Table 3. Child characteristics and unplanned placement change rates**

All children's homes placements that started between 01/04/2019 and 31/03/2023 where the risk period of a placement change is at least one year. Placements starting for children aged 17 who exited care within one year (due to aging-out of care) and other early exits from care were excluded. The data is restricted to placements starting before 31/03/2023 but full follow-up (for the outcome of a placement change) is known up until 31/03/2024 across all cases. Columns highlighted in yellow are mutually exclusive and show the percentage of placement changes that were 'unplanned', 'planned', and 'other'. Columns highlighted in green provide a finer breakdown of the unplanned placement changes and shows the percentage of placement changes that were made due to the following reasons: 1) provider requests end due to child’s behaviour; 2) Provider requests end for any other reason; 3) Child requests end; 4) LA requests end.

Underlying codes / groupings are shown in Table 5.

| **Characteristic** | **Any placement change** | **Placement change type** | | | | | | |
| --- | --- | --- | --- | --- | --- | --- | --- | --- |
|  |  | **Unplanned placement change** | **Planned placement change** | **Other** | **Breakdown of unplanned placement changes** | | | |
|  |  |  |  |  | **Provider requests end due to child’s behaviour** | **Provider requests end for any other reason** | **Child requests end** | **LA requests end** |
| Total (16,360) | 49.7% | **19.1%** | **18.2%** | **12.4%** | **12.2%** | **3.1%** | **1.8%** | **2.0%** |
| **Demographics** |  |  |  |  |  |  |  |  |
| Age when placement started |  |  |  |  |  |  |  |  |
| 12 and under (4,430) | 37.3% | **13.7%** | **15.0%** | **8.6%** | **8.7%** | **2.5%** | **0.5%** | **2.0%** |
| 13 to 14 (5,960) | 49.6% | **20.1%** | **17.3%** | **12.1%** | **13.3%** | **3.3%** | **1.5%** | **2.0%** |
| 15 to 16 (6,130) | 58.8% | **21.9%** | **21.4%** | **15.5%** | **13.5%** | **3.3%** | **3.1%** | **2.0%** |
| Gender |  |  |  |  |  |  |  |  |
| Males (9,600) | 47.6% | **18.6%** | **17.3%** | **11.8%** | **12.0%** | **3.2%** | **1.6%** | **1.8%** |
| Females (6,920) | 52.6% | **19.8%** | **19.5%** | **13.3%** | **12.3%** | **3.0%** | **2.2%** | **2.3%** |
| Ethnicity |  |  |  |  |  |  |  |  |
| Asian (450) | 52.1% | **14.1%** | **24.0%** | **13.9%** | **8.3%** | **2.9%** | **1.6%** | **1.3%** |
| Black (1,130) | 56.5% | **20.3%** | **24.7%** | **11.5%** | **13.6%** | **3.4%** | **1.4%** | **1.9%** |
| Mixed (1,710) | 55.2% | **24.3%** | **18.1%** | **12.8%** | **15.4%** | **4.0%** | **2.2%** | **2.7%** |
| Other (240) | 43.3% | **10.5%** | **22.9%** | **10.0%** | **7.1%** | **0.8%** | **1.3%** | **1.3%** |
| White (12,990) | 48.5% | **18.6%** | **17.4%** | **12.4%** | **11.8%** | **3.0%** | **1.8%** | **2.0%** |
| **Prior care characteristics** |  |  |  |  |  |  |  |  |
| Prior placement history |  |  |  |  |  |  |  |  |
| No prior placement (2,890) | 44.4% | **12.7%** | **18.8%** | **12.8%** | **7.4%** | **2.5%** | **1.1%** | **1.7%** |
| Prior CH \| planned \| 1 to 3 placements (1,340) | 50.1% | **17.2%** | **19.1%** | **13.7%** | **11.8%** | **2.2%** | **1.9%** | **1.3%** |
| Prior CH \| planned \| 4+ placements (1,790) | 55.6% | **17.3%** | **21.0%** | **17.3%** | **11.4%** | **2.2%** | **2.0%** | **1.7%** |
| Prior CH \| unplanned \| 1 to 3 placements (880) | 57.7% | **30.8%** | **17.0%** | **9.9%** | **21.8%** | **4.8%** | **1.5%** | **2.7%** |
| Prior CH \| unplanned \| 4+ placements (1,180) | 63.7% | **36.6%** | **18.3%** | **8.8%** | **24.9%** | **5.5%** | **2.5%** | **3.7%** |
| Prior FC \| planned \| 1 to 3 placements (1,660) | 42.5% | **11.5%** | **19.0%** | **11.8%** | **6.4%** | **2.3%** | **0.8%** | **2.0%** |
| Prior FC \| planned \| 4+ placements (1,570) | 45.8% | **13.6%** | **16.9%** | **15.3%** | **8.9%** | **1.9%** | **1.4%** | **1.4%** |
| Prior FC \| unplanned \| 1 to 3 placements (1,580) | 42.0% | **19.9%** | **15.0%** | **7.0%** | **11.6%** | **3.4%** | **2.6%** | **2.3%** |
| Prior FC \| unplanned \| 4+ placements (1,300) | 41.6% | **20.2%** | **14.6%** | **6.9%** | **12.3%** | **4.3%** | **1.7%** | **1.9%** |
| Prior other \| planned \| 1 to 3 placements (910) | 50.3% | **17.5%** | **19.2%** | **13.6%** | **11.3%** | **2.8%** | **1.9%** | **1.5%** |
| Prior other \| planned \| 4+ placements (1,010) | 65.5% | **25.6%** | **20.6%** | **19.3%** | **16.9%** | **3.6%** | **3.5%** | **1.6%** |
| Prior other \| unplanned \| 1 to 3 placements (170) | 55.8% | **26.8%** | **20.3%** | **8.7%** | **15.7%** | **4.1%** | **2.9%** | **4.1%** |
| Prior other \| unplanned \| 4+ placements (250) | 67.3% | **38.9%** | **17.7%** | **10.6%** | **23.6%** | **6.3%** | **4.3%** | **4.7%** |
| Prior periods of care |  |  |  |  |  |  |  |  |
| None (12,470) | 47.9% | **18.4%** | **17.6%** | **11.8%** | **11.6%** | **3.1%** | **1.7%** | **2.0%** |
| 1 (3,040) | 54.8% | **21.1%** | **19.8%** | **13.9%** | **14.0%** | **3.0%** | **2.0%** | **2.1%** |
| 2+ (840) | 59.2% | **21.9%** | **21.2%** | **16.1%** | **13.6%** | **3.4%** | **2.5%** | **2.4%** |
| Assessment factors prior to CH placement |  |  |  |  |  |  |  |  |
| Domestic abuse and violence (410) | 46.9% | **18.8%** | **18.1%** | **9.9%** | **12.8%** | **2.9%** | **1.4%** | **1.7%** |
| Complexities around parental mental hea (740) | 46.5% | **16.2%** | **19.4%** | **10.9%** | **10.2%** | **2.2%** | **1.9%** | **1.9%** |
| Disability (1,270) | 32.7% | **10.2%** | **13.7%** | **8.7%** | **6.1%** | **2.0%** | **0.9%** | **1.2%** |
| Risks outside the home (1,290) | 66.8% | **27.0%** | **24.4%** | **15.4%** | **16.8%** | **4.8%** | **2.7%** | **2.7%** |
| Complex domestic abuse / risks at home (1,280) | 41.5% | **16.1%** | **15.8%** | **9.7%** | **10.6%** | **2.6%** | **1.0%** | **1.9%** |
| Childs mental health (1,710) | 50.3% | **20.3%** | **16.2%** | **13.8%** | **13.3%** | **3.4%** | **1.8%** | **1.8%** |
| Physical abuse (300) | 45.0% | **17.3%** | **15.7%** | **12.0%** | **10.0%** | **1.7%** | **1.3%** | **4.3%** |
| Neglect (240) | 52.7% | **14.5%** | **28.2%** | **10.0%** | **8.7%** | **2.5%** | **0.8%** | **2.5%** |
| Concerns about another person (310) | 40.4% | **15.1%** | **12.5%** | **12.8%** | **7.7%** | **2.9%** | **1.9%** | **2.6%** |
| Sexual abuse (150) | 41.8% | **15.8%** | **20.9%** | **5.2%** | **12.4%** | **0.7%** | **0.7%** | **2.0%** |
| Risks in and outside the home (1,990) | 61.9% | **27.0%** | **17.8%** | **17.4%** | **17.7%** | **4.0%** | **2.7%** | **2.6%** |
| Other (700) | 49.1% | **18.1%** | **18.9%** | **12.2%** | **11.5%** | **1.9%** | **2.1%** | **2.6%** |
| No assessment within two years of placement starting (6,130) | 48.7% | **18.0%** | **18.8%** | **11.8%** | **11.4%** | **3.1%** | **1.8%** | **1.7%** |
| CP plan within two years of period of care starting |  |  |  |  |  |  |  |  |
| No (13,180) | 49.8% | **19.0%** | **18.2%** | **12.6%** | **12.2%** | **3.0%** | **1.9%** | **1.9%** |
| Yes (3,350) | 49.4% | **19.5%** | **18.1%** | **11.8%** | **12.1%** | **3.5%** | **1.6%** | **2.3%** |
| **Care and provider characteristics at entry to placement** |  |  |  |  |  |  |  |  |
| Legal status at entry to CH |  |  |  |  |  |  |  |  |
| ICO (2,810) | 49.8% | **19.6%** | **18.4%** | **11.9%** | **12.8%** | **3.0%** | **1.4%** | **2.4%** |
| FCO (8,620) | 50.2% | **20.4%** | **17.7%** | **12.1%** | **12.9%** | **3.3%** | **2.3%** | **1.9%** |
| Police / LA (140) | 71.9% | **15.8%** | **33.8%** | **22.3%** | **10.8%** | **3.6%** | **0.7%** | **0.7%** |
| S20 (4,960) | 48.3% | **16.7%** | **18.6%** | **13.0%** | **10.6%** | **2.8%** | **1.3%** | **2.0%** |
| Distance placed and LA |  |  |  |  |  |  |  |  |
| Within LA \| In-house (3,000) | 62.6% | **11.6%** | **30.4%** | **20.6%** | **5.3%** | **2.2%** | **1.9%** | **2.2%** |
| Within LA \| Outsourced (2,970) | 43.3% | **19.3%** | **13.8%** | **10.1%** | **12.8%** | **3.0%** | **1.9%** | **1.6%** |
| Out of LA \| Outsourced \| Less than 20 miles (3,370) | 46.0% | **21.1%** | **15.1%** | **9.8%** | **13.4%** | **3.8%** | **1.8%** | **2.1%** |
| Out of LA \| Outsourced \| 20 to 50 miles (3,100) | 47.6% | **23.3%** | **14.3%** | **10.0%** | **15.9%** | **3.3%** | **2.0%** | **2.1%** |
| Out of LA \| Outsourced \| More than 50 miles (4,080) | 49.7% | **19.6%** | **18.0%** | **12.1%** | **12.9%** | **3.0%** | **1.7%** | **2.0%** |
| Maximum number of occupants during placement |  |  |  |  |  |  |  |  |
| 1 (2,490) | 59.1% | **24.5%** | **20.2%** | **14.5%** | **16.7%** | **3.7%** | **1.7%** | **2.4%** |
| 2 (3,520) | 51.7% | **20.9%** | **17.6%** | **13.0%** | **13.6%** | **3.1%** | **2.2%** | **2.0%** |
| 3 (3,860) | 48.9% | **19.0%** | **18.0%** | **11.8%** | **12.0%** | **3.2%** | **2.0%** | **1.8%** |
| 4 (3,000) | 49.7% | **18.5%** | **17.9%** | **13.3%** | **11.2%** | **3.1%** | **1.8%** | **2.4%** |
| 5 (1,690) | 49.6% | **17.5%** | **19.8%** | **12.3%** | **11.0%** | **3.0%** | **1.6%** | **1.9%** |
| 6+ (1,960) | 36.1% | **11.3%** | **16.2%** | **8.6%** | **6.7%** | **2.2%** | **1.1%** | **1.3%** |
| Number of beds (based on closest in-year provider snapshot after child's entry to CH) |  |  |  |  |  |  |  |  |
| 1 to 3 beds (5,100) | 48.6% | **22.2%** | **15.8%** | **10.6%** | **14.7%** | **3.4%** | **2.2%** | **1.9%** |
| 4 to 6 beds (9,050) | 53.4% | **19.1%** | **19.9%** | **14.5%** | **11.9%** | **3.1%** | **1.9%** | **2.2%** |
| 7+ beds (2,380) | 38.1% | **12.8%** | **17.1%** | **8.3%** | **7.7%** | **2.6%** | **1.0%** | **1.5%** |
| Registered manager in place at entry to placement (new manager registered within 90 days) |  |  |  |  |  |  |  |  |
| Yes (11,690) | 48.4% | **18.4%** | **17.7%** | **12.3%** | **11.7%** | **3.0%** | **1.7%** | **2.0%** |
| No (4,840) | 52.9% | **20.8%** | **19.4%** | **12.8%** | **13.2%** | **3.3%** | **2.2%** | **2.1%** |
| Turnover and agency worker use (data for post-2019 only) |  |  |  |  |  |  |  |  |
| 0% to 25% turnover (3,800) | 49.0% | **17.7%** | **18.2%** | **13.0%** | **10.8%** | **3.3%** | **1.8%** | **1.8%** |
| 25% to 50% turnover (2,300) | 45.8% | **20.2%** | **14.2%** | **11.5%** | **12.5%** | **3.5%** | **2.0%** | **2.2%** |
| More than 50% turnover (2,300) | 51.0% | **20.0%** | **19.7%** | **11.2%** | **12.8%** | **3.2%** | **2.0%** | **2.0%** |
| 0% to 25% turnover + use of agency staff (3,620) | 52.4% | **16.5%** | **22.0%** | **13.9%** | **10.6%** | **2.3%** | **1.8%** | **1.8%** |
| 25% to 50% turnover + use of agency staff (2,200) | 45.9% | **18.2%** | **16.7%** | **11.0%** | **11.6%** | **3.0%** | **1.5%** | **2.1%** |
| More than 50% turnover + use of agency staff (2,310) | 53.0% | **24.1%** | **16.3%** | **12.6%** | **16.4%** | **3.6%** | **1.9%** | **2.2%** |
| CH tenure at placement start |  |  |  |  |  |  |  |  |
| Less than 1 year (3,050) | 51.8% | **23.5%** | **17.1%** | **11.2%** | **16.7%** | **3.3%** | **1.5%** | **2.0%** |
| 1 to 4 years (4,590) | 49.5% | **20.1%** | **17.5%** | **12.1%** | **12.3%** | **3.4%** | **2.0%** | **2.4%** |
| 5 to 9 years (3,440) | 49.1% | **19.7%** | **17.2%** | **12.2%** | **12.6%** | **3.3%** | **2.0%** | **1.8%** |
| 10+ years (5,440) | 49.1% | **15.6%** | **20.1%** | **13.5%** | **9.2%** | **2.6%** | **1.9%** | **1.9%** |

**Table 4. Predicted probability of an unplanned placement change based on multinomial regression model**

All children's homes placements that started between 01/04/2019 and 31/03/2023 where the risk period of a placement change is at least one year. Placements starting for children aged 17 who exited care within one year (due to aging-out of care) and other early exits from care were excluded. The data is restricted to placements starting before 31/03/2023 but full follow-up (for the outcome of a placement change) is known up until 31/03/2024 across all cases.

Model 1 shows the predicted probability of an unplanned placement change (based on the multinomial regression model) including only demographics variables; Model 2 includes demographic and prior care characteristic variables; Model 3 includes demographic, prior care characteristic, and care/provider characteristics at entry.

| **Characteristic** | **Unplanned** | | | | | |
| --- | --- | --- | --- | --- | --- | --- |
|  | **Model 1** | | **Model 2** | | **Model 3** | |
|  | **RR** | **CI** | **RR** | **CI** | **RR** | **CI** |
|  |  |  |  |  |  |  |
| **Demographics** |  |  |  |  |  |  |
| Age when placement started |  |  |  |  |  |  |
| 12 and under (4,430) | 13.7% | 12.7% to 14.7% | 15.5% | 14.4% to 16.6% | 15.6% | 14.5% to 16.8% |
| 13 to 14 (5,960) | 20.1% | 19.1% to 21.1% | 19.8% | 18.9% to 20.8% | 19.6% | 18.7% to 20.6% |
| 15 to 16 (6,130) | 22.1% | 21.0% to 23.1% | 20.9% | 19.9% to 21.9% | 21.1% | 20.1% to 22.1% |
| Gender |  |  |  |  |  |  |
| Males (9,600) | 19.0% | 18.2% to 19.8% | 19.3% | 18.5% to 20.1% | 19.5% | 18.7% to 20.3% |
| Females (6,920) | 19.3% | 18.4% to 20.2% | 18.9% | 18.0% to 19.8% | 18.7% | 17.8% to 19.5% |
| Ethnicity |  |  |  |  |  |  |
| Asian (450) | 13.7% | 10.6% to 16.9% | 15.4% | 12.0% to 18.8% | 16.2% | 12.7% to 19.7% |
| Black (1,130) | 19.7% | 17.4% to 21.9% | 19.8% | 17.5% to 22.0% | 19.7% | 17.5% to 22.0% |
| Mixed (1,710) | 24.4% | 22.4% to 26.4% | 24.0% | 22.0% to 25.9% | 23.7% | 21.8% to 25.6% |
| Other (240) | 10.4% | 6.5% to 14.2% | 11.8% | 7.6% to 16.1% | 12.6% | 8.2% to 17.1% |
| White (12,990) | 18.7% | 18.0% to 19.4% | 18.6% | 18.0% to 19.3% | 18.6% | 18.0% to 19.3% |
| **Prior care characteristics** |  |  |  |  |  |  |
| Prior placement history |  |  |  |  |  |  |
| No prior placement (2,890) |  |  | 13.0% | 11.7% to 14.2% | 13.2% | 11.7% to 14.6% |
| Prior CH \| planned \| 1 to 3 placements (1,340) |  |  | 16.8% | 14.8% to 18.8% | 16.7% | 14.8% to 18.7% |
| Prior CH \| planned \| 4+ placements (1,790) |  |  | 17.1% | 15.4% to 18.8% | 17.2% | 15.4% to 18.9% |
| Prior CH \| unplanned \| 1 to 3 placements (880) |  |  | 29.4% | 26.4% to 32.4% | 28.3% | 25.4% to 31.2% |
| Prior CH \| unplanned \| 4+ placements (1,180) |  |  | 35.8% | 33.1% to 38.5% | 35.2% | 32.4% to 38.0% |
| Prior FC \| planned \| 1 to 3 placements (1,660) |  |  | 11.9% | 10.3% to 13.5% | 12.0% | 10.4% to 13.5% |
| Prior FC \| planned \| 4+ placements (1,570) |  |  | 14.5% | 12.7% to 16.3% | 14.7% | 12.9% to 16.5% |
| Prior FC \| unplanned \| 1 to 3 placements (1,580) |  |  | 21.1% | 19.1% to 23.2% | 21.5% | 19.5% to 23.6% |
| Prior FC \| unplanned \| 4+ placements (1,300) |  |  | 22.2% | 19.8% to 24.5% | 22.8% | 20.3% to 25.2% |
| Prior other \| planned \| 1 to 3 placements (910) |  |  | 16.1% | 13.8% to 18.4% | 15.4% | 13.2% to 17.6% |
| Prior other \| planned \| 4+ placements (1,010) |  |  | 23.9% | 21.3% to 26.5% | 23.3% | 20.8% to 25.9% |
| Prior other \| unplanned \| 1 to 3 placements (170) |  |  | 24.3% | 18.1% to 30.5% | 23.6% | 17.6% to 29.6% |
| Prior other \| unplanned \| 4+ placements (250) |  |  | 36.0% | 30.3% to 41.8% | 35.2% | 29.5% to 40.8% |
| Prior periods of care |  |  |  |  |  |  |
| None (12,470) |  |  | 18.7% | 18.1% to 19.4% | 18.8% | 18.1% to 19.4% |
| 1 (3,040) |  |  | 20.1% | 18.8% to 21.5% | 20.1% | 18.7% to 21.4% |
| 2+ (840) |  |  | 20.9% | 18.3% to 23.5% | 21.0% | 18.4% to 23.7% |
| Assessment factors prior to CH placement |  |  |  |  |  |  |
| Domestic abuse and violence (410) |  |  | 20.1% | 16.2% to 23.9% | 20.0% | 16.2% to 23.9% |
| Complexities around parental mental hea (740) |  |  | 17.1% | 14.3% to 19.8% | 17.7% | 14.9% to 20.5% |
| Disability (1,270) |  |  | 12.3% | 10.4% to 14.2% | 12.7% | 10.7% to 14.7% |
| Risks outside the home (1,290) |  |  | 25.8% | 23.4% to 28.1% | 25.5% | 23.2% to 27.9% |
| Complex domestic abuse / risks at home (1,280) |  |  | 17.5% | 15.3% to 19.6% | 17.1% | 15.0% to 19.2% |
| Childs mental health (1,710) |  |  | 20.8% | 18.9% to 22.7% | 20.4% | 18.5% to 22.3% |
| Physical abuse (300) |  |  | 18.8% | 14.3% to 23.3% | 18.5% | 14.1% to 22.9% |
| Neglect (240) |  |  | 15.7% | 11.1% to 20.4% | 16.0% | 11.3% to 20.7% |
| Concerns about another person (310) |  |  | 17.0% | 12.7% to 21.4% | 16.7% | 12.4% to 20.9% |
| Sexual abuse (150) |  |  | 16.3% | 10.4% to 22.1% | 15.8% | 10.1% to 21.5% |
| Risks in and outside the home (1,990) |  |  | 25.6% | 23.7% to 27.5% | 25.1% | 23.2% to 26.9% |
| Other (700) |  |  | 17.8% | 15.0% to 20.6% | 17.8% | 15.0% to 20.5% |
| No assessment within two years of placement starting (6,130) |  |  | 17.4% | 16.4% to 18.3% | 17.6% | 16.7% to 18.6% |
| CP plan within two years of period of care starting |  |  |  |  |  |  |
| No (13,180) |  |  | 19.1% | 18.4% to 19.8% | 19.2% | 18.5% to 19.9% |
| Yes (3,350) |  |  | 19.1% | 17.8% to 20.5% | 18.7% | 17.3% to 20.1% |
| **Care and provider characteristics at entry to placement** |  |  |  |  |  |  |
| Legal status at entry to CH |  |  |  |  |  |  |
| ICO (2,810) |  |  |  |  | 19.9% | 18.4% to 21.5% |
| FCO (8,620) |  |  |  |  | 18.6% | 17.7% to 19.4% |
| Police / LA (140) |  |  |  |  | 21.2% | 13.7% to 28.6% |
| S20 (4,960) |  |  |  |  | 19.7% | 18.3% to 21.0% |
| Distance placed and LA |  |  |  |  |  |  |
| Within LA \| In-house (3,000) |  |  |  |  | 12.6% | 11.3% to 13.9% |
| Within LA \| Outsourced (2,970) |  |  |  |  | 20.4% | 18.9% to 21.8% |
| Out of LA \| Outsourced \| Less than 20 miles (3,370) |  |  |  |  | 20.7% | 19.4% to 22.1% |
| Out of LA \| Outsourced \| 20 to 50 miles (3,100) |  |  |  |  | 22.3% | 20.9% to 23.7% |
| Out of LA \| Outsourced \| More than 50 miles (4,080) |  |  |  |  | 19.0% | 17.8% to 20.2% |
| Maximum number of occupants during placement |  |  |  |  |  |  |
| 1 (2,490) |  |  |  |  | 21.9% | 20.2% to 23.6% |
| 2 (3,520) |  |  |  |  | 20.1% | 18.8% to 21.4% |
| 3 (3,860) |  |  |  |  | 18.8% | 17.6% to 20.0% |
| 4 (3,000) |  |  |  |  | 18.8% | 17.4% to 20.3% |
| 5 (1,690) |  |  |  |  | 18.5% | 16.6% to 20.4% |
| 6+ (1,960) |  |  |  |  | 14.0% | 12.2% to 15.9% |
| Number of beds (based on closest in-year provider snapshot after child's entry to CH) |  |  |  |  |  |  |
| 1 to 3 beds (5,100) |  |  |  |  | 18.4% | 17.3% to 19.5% |
| 4 to 6 beds (9,050) |  |  |  |  | 19.7% | 18.9% to 20.6% |
| 7+ beds (2,380) |  |  |  |  | 18.2% | 16.2% to 20.2% |
| Registered manager in place at entry to placement (new manager registered within 90 days) |  |  |  |  |  |  |
| Yes (11,690) |  |  |  |  | 18.6% | 17.9% to 19.3% |
| No (4,840) |  |  |  |  | 20.4% | 19.3% to 21.5% |
| Turnover and agency worker use (data for post-2019 only) |  |  |  |  |  |  |
| 0% to 25% turnover (3,800) |  |  |  |  | 17.6% | 16.5% to 18.8% |
| 25% to 50% turnover (2,300) |  |  |  |  | 19.3% | 17.7% to 20.8% |
| More than 50% turnover (2,300) |  |  |  |  | 18.2% | 16.8% to 19.7% |
| 0% to 25% turnover + use of agency staff (3,620) |  |  |  |  | 18.9% | 17.6% to 20.2% |
| 25% to 50% turnover + use of agency staff (2,200) |  |  |  |  | 18.9% | 17.3% to 20.6% |
| More than 50% turnover + use of agency staff (2,310) |  |  |  |  | 22.6% | 21.0% to 24.2% |
| CH tenure at placement start |  |  |  |  |  |  |
| Less than 1 year (3,050) |  |  |  |  | 20.3% | 18.9% to 21.8% |
| 1 to 4 years (4,590) |  |  |  |  | 19.0% | 17.9% to 20.1% |
| 5 to 9 years (3,440) |  |  |  |  | 19.1% | 17.8% to 20.4% |
| 10+ years (5,440) |  |  |  |  | 18.3% | 17.1% to 19.4% |

**Table 5. Model metrics**

| **Model metrics** | **Model 1** | **Model 2** | **Model 3** |
| --- | --- | --- | --- |
| AIC | 40106.9 | 39119.1 | 38148.2 |
| BIC | 40292 | 39928.9 | 39490.2 |
| McFadden’s pseudo R^2^ | 0.0154 | 0.0437 | 0.0709 |

Notes:

McFadden’s pseudo R² represents improvement in model fit relative to the intercept-only model and should not be interpreted as a proportion of explained variance.

Lower AIC/BIC indicates improved fit across models

**Table 6. Coding for placement changes**

| **Placement change codes** | **Placement change description** | **Coding for placement changes as 3 groups** | **Coding for placement changes as 6 groups** |  |
| --- | --- | --- | --- | --- |
|  |  |  |  |  |
|  |  |  |  |  |
| CREQB | Carer(s) requests placement end due to child’s behaviour | Unplanned | Provider requests end due to childs behaviour |  |
| CREQO | Carer(s) requests placement end other than due to child’s behaviour | Unplanned | Provider requests end for any other reason |  |
| CLOSE | Resignation/ closure of provision | Unplanned | Provider requests end for any other reason |  |
| CHILD | Child requests placement end | Unplanned | Child requests end |  |
| ALLEG | Allegation (s47) | Unplanned | LA requests end |  |
| STAND | Standards of care concern | Unplanned | LA requests end |  |
| APPRR | Approval removed | Unplanned | LA requests end |  |
| LAREQ | Responsible/area authority requests placement end | Unplanned | LA requests end |  |
| CARPL | Change to/Implementation of Care Plan | Planned | Move planned as part of childs care plan |  |
| CUSTOD | Custody arrangement | Other | Other |  |
| OTHER | Other | Other | Other |  |
